# Stress Perceived by University Health Sciences Students, 1 Year after COVID-19 Pandemic

**DOI:** 10.3390/ijerph18105233

**Published:** 2021-05-14

**Authors:** Yolanda Marcén-Román, Angel Gasch-Gallen, Irene Isabel Vela Martín de la Mota, Estela Calatayud, Isabel Gómez-Soria, Beatriz Rodríguez-Roca

**Affiliations:** 1 Faculty of Health Sciences, University of Zaragoza, 50009 Zaragoza, Spain; yomarcen@unizar.es (Y.M.-R.); angelgasch@unizar.es (A.G.-G.); Brodriguez@unizar.es (B.R.-R.); 2 Research Group Physiotherapy (GIIS086), Institute of Research of Aragon, 50009 Zaragoza, Spain; 3 Urology Department, University Clinical Hospital, 50009 Zaragoza, Spain; irenica_bis@hotmail.com

**Keywords:** stress, COVID-19, university health students, anxiety, depression

## Abstract

Today’s COVID-19 situation can affect university Health Sciences students’ psychological health. This study aimed to analyze the stress caused by the impact of the COVID-19 pandemic on Health Sciences students from the University of Zaragoza (Spain) almost 1 year after the pandemic began. This cross-sectional descriptive study was conducted with a sample of 252 university students who completed a self-administered online questionnaire. It evaluated the impact of perceived stress with a modified scale (PSS-10-C), and assessed anxiety and depression on the Goldberg scale. Students presented stress (13.1%), anxiety (71.4%) and depression (81%). Females (81.7%) and the third-year Occupational Therapy students (*p* = 0.010) reported perceived stress. Nursing students perceived less stress (OR: 0.148; 95% CI: 0.026 to 0.842). University students developed stress and anxiety due to COVID-19 almost 1 year after the pandemic began. Psychological support measures for these groups should be prioritized.

## 1. Introduction

The COVID-19 infectious disease pandemic caused by SARS-COV2 is a serious public health problem [1]. Although each country has adopted specific strategies to avoid this virus spreading, social distancing and teleworking have been the general measures to achieve this [2]. On 14 March 2020, Spain declared a national state of alarm when non-essential jobs were not carried out, schools and universities closed and the population was confined at home. This pandemic has changed our way of life, as well as university students’ academic training partly because faculties being closed for long periods, and a shift toward technology-based learning and social isolation took place during the quarantine imposed by the State [3,4,5].

Recent literature reviews conclude that quarantine measures could have negative psychological effects [6,7,8,9,10,11,12,13,14], including symptoms of post-traumatic stress, anxiety and depression [15]. Stress is the most frequently detected problem in this uncertainty context, along with fear of transmitting the virus, followed by psychological diseases [16,17]. In this situation, university students report high levels of stress, anxiety and depression [18,19,20], which affects student motivation and their attitudes to learning [21,22].

Different studies have dealt with the training of future health professionals in these circumstances as their training had to be intensely and quickly adapted due to organizational and academic changes [23]. Although it has been an important challenge for students in general [24], current research sheds light on specific learner typologies, such as those in full-time jobs, those who are financially independent, or caregivers [25], by highlighting the usefulness of methodologies that focus on reducing spent time and unvaluable activities applied to online education, specifically during the COVID-19 pandemic [26].

Another point raised is that the quantity and frequency of social interactions might be an indicator of clinical predictions of social anxiety, isolation, depression and stress [27].

Although some specific instruments measure emotional well-being during quarantine, information on the psychometric properties of these scales is still scarce. We used the Perceived Stress Scale (PSS-10), modified for COVID-19 (PSS-10-C), because this instrument’s internal consistency is acceptable, as other studies have shown [28,29,30,31], and it has been employed in studies that measure students’ stress [32,33,34,35].

Despite different works identifying that the general university’s population mental health has altered as reflected by today’s pandemic and the negative effect that confinement has on student learning [36,37], the impact of this pandemic on university education in general, and on Health Sciences students after confinement in particular, remains unknown.

This study aimed to analyze the stress caused by the COVID-19 pandemic perceived by university Health Sciences students from the University of Zaragoza, along with their socio-demographic characteristics, anxiety and depression after almost 1 year of the COVID-19 pandemic.

## 2. Materials and Methods

A cross-sectional descriptive study was performed of the association between the perceived stress caused by the COVID-19 pandemic and the characteristics of students studying Health Sciences Degrees.

### 2.1. Participants

Information was collected from the students of the nursing, physiotherapy and occupational therapy degrees at the health sciences faculty of the University of Zaragoza. The inclusion criterion was to answer all of the ‘stress by covid’ questionnaire (PSS-10-C). The two exclusion criteria were not studying any of these degrees and not understanding Spanish.

A sample size of 252 students was estimated by taking a 4.65% error margin for proportions for frequency of results on these scales, as calculated by the EPIDAT 4.2 program. As a maximum 5% margin error is recommended, we believe that obtaining these data would mean that the population size of our study was big enough.

### 2.2. Instruments

In order to know students’ psychological status, the questionnaire on stress caused by COVID-19 [29] and the Goldberg abbreviated anxiety and depression scale (GADS) [38,39], were employed. To measure perceived stress caused by COVID-19, the modified PSS-10 version related to COVID-19 (PSS-10-C) was applied. It comprises 10 items. Each offers five response options: “never”, “almost never”, “occasionally”, “almost always”, “always”. They are classified from 0 to 4. Items 1, 2, 3, 6, 9 and 10 are directly scored from 0 to 4. Conversely, items 4, 5, 7 and 8 are scored from 4 to 0. The higher the score, the more the perceived stress. A cutoff point of ≥25 is related to high perceived stress for COVID-19, [40]. The abbreviated GADS measures anxiety and depression in the general population by examining four basic psychiatric areas: depression, anxiety, social anxiety disorder, hypochondria. This instrument has been previously validated and combines demonstrated applicability qualities. Cutoff points equal or exceed 4 for the anxiety score, and equal or exceed 2 for depression. Sensitivity (83.0%) and specificity (81.8%) give a 95% positive predictive value [39].

### 2.3. Procedure

An online questionnaire was designed and later diffused by a web link to different student media in January 2021. Participation in this study was completely anonymous and voluntary.

This study included the following socio-demographic variables: gender (males/female), age, degree (nursing, physiotherapy, occupational therapy), academic year (first, second, third, fourth), occupational status (working fulltime, working part-time, doing unpaid work, unpaid voluntary work), civil status, economic situation, place of residence (urban/rural) and country born in (Spain/elsewhere).

This study was performed according to the Declaration of Helsinki. Data were confidentially processed in line with Spanish Organic Law LOPD 03/2018 on Personal Data Protection. Consent came from the Committee of Research Ethics of the Spanish Autonomous Aragón Community (CEICA) before the study began (Ref: C.P.-C.I. PI21/004).

### 2.4. Statistical Analysis

A descriptive analysis was performed with frequency and percentages for the qualitative variables and with mean + standard deviation for the quantitative variables. To compare the quantitative variables, the Student’s *t*-test was used, or the corresponding non-parametric tests were run for those variables not following normal distribution. Pearson’s or Spearman’s (Rho) correlation coefficient was studied to examine the correlation among variables. A bivariate study was performed to determine the dependence between two categorical variables by applying the statistical χ^2^ test. Logistic regression models were built to study the relation between the risk of depression and the different study variables. These models included variables showing a statistical significance of *p* < 0.2 in the bivariate study. The significance level of all the analyses was set at *p* ≤ 0.05. The quantitative data were analyzed using SPSS, version 26 (IBM, Corp., Chicago, IL, USA).

## 3. Results

Two hundred to fifty-two health sciences students participated in this study one year after the start of the pandemic.

The participants’ mean age was 21.02 years (SD 5.20) and 81.7% were female. The majority were born in Spain (92.1%). The following data were obtained: 74.6% lived in an urban area, 72.6% had never smoked, 84.5% had no chronic disease, 78.2% depended on family income and 8.8% did unpaid work, 89.7% did not have COVID-19 and 80.2% reported no family relative with COVID-19. Due to the COVID-19 pandemic, 71.4% had anxiety, 81% depression and 13.1% stress. Table 1 shows the general sample’s characteristics.

Table 2 shows the correlations between the employed psychological scales. A positive correlation appeared among perceived stress due to COVID-19, anxiety and depression, with a stronger correlation between anxiety and depression. The students with more stress were associated with being at higher risk of developing anxiety and depression. The employed scales conferred the study reliability because they obtained a score over 0.7: the Stress PSS-10-C (0.789) and GADS anxiety and depression (0.794 vs. 0.752).

Those students with stress were mostly female (90.9%) studied in year 3 of the occupational therapy degree (*p* = 0.010) and 81.8% did not work (*p* = 0.038). Of the students with stress, 90.9% also developed anxiety (*p* = 0.013). Those students who had a relative with COVID-19 presented less stress, although 82.2% of the students stated that they had no family relative with this disease (Table 3).

When studying university degrees, we found that nursing students had less perceived stress (OR: 0.148; 95% CI: 0.026 to 0.842) than the third-year students of the two other health sciences degrees. Those with anxiety were 1.5-folder more likely to perceive considerable stress due to COVID-19 (OR: 1.504; 95% CI: 1.228–1.842). The students who indicated having a relative with COVID-19 were at less risk of suffering stress (OR: 0.042; 95% CI: 146 to 0.967) (Table 4).

## 4. Discussion

This study analyzed University of Zaragoza health sciences students’ perceived stress due to the impact of the COVID-19 pandemic.

Almost 1 year after the pandemic started, our study obtained data which showed that 13.1% of the university students from all three degrees perceived stress. These data were lower than the figures reported by Odriozola et al., [11] when the pandemic began, who indicated that 28.14% of those surveyed reported stress symptoms. This could be because our students had more information about COVID-19 and a more stabilized academic situation.

The third-year occupational therapy students perceived more stress (50%). We believe this was because they went on placements in old people’s homes, which were very seriously affected by COVID-19 during the pandemic [41]. The third-year physiotherapy students, and significantly the nursing students, perceived less stress at only 10% and 40% respectively. This could be because, despite almost all their curricular placements taking place in hospitals, they did not directly work with COVID-19 patients. Nevertheless, some studies [42,43] indicate that final-year students perceived more stress because their future work was academically uncertain and unsure.

GADS indicated that 71.4% of the students had anxiety and 81% had depression almost 1 year after the pandemic emerged. One previous study performed with a Chinese population indicated that younger people aged under 35 years were at more risk of suffering anxiety and stress than older people [44]. Some evidence suggests that stress is related to anxiety and depression [45], and our study demonstrated an association between perceived stress and anxiety.

The data from studies in China differ. Cao et al., [46] reported that 24.9% of students’ mental health was affected by anxiety, while Tang et al., [47] indicated 1 month after the pandemic commenced that 2.7% of students had post-traumatic stress and 9% presented depressive symptoms. This difference in data could be due to the distinct speed at which the virus spread between provinces. In Pakistan, one work indicated that 34% of students reported moderate to severe anxiety symptoms [48]. Their data are similar to those obtained in a study conducted in Turkey to explore anxiety-related symptoms (45%) [49]. Higher levels of anxiety and depression were found in Cyprus, respectively, with 64.1% and 57.3% [50].

Of all our students, 90.9% who had stress were female. This coincides with other studies [8,13,49,51,52]. However, this was not significant, perhaps because it included a only a small number of males. Some pieces of evidence reveal that females report more fear and are more likely to develop anxiety disorders than males [53], who are associated with more vulnerability deriving from mental disorders, specifically with females [54]. All these factors could lead to more perceived stress.

We explored several demographic and contextual factors, e.g., a correlation of symptoms, including occupational status, because COVID-19 had not proportionally affected families, and financial difficulties can affect mental health. Our data reflected that the stress perceived by the students who did not work (81.8%) was higher than those who worked, perhaps because unemployment and not being able to cover monthly expenses during the pandemic are associated with more psychological anguish in young adults [55].

Of all our students, 44.4% had been in quarantine given the risk of catching the virus from family relatives testing positive for COVID-19. Of these, 10.3% had caught the virus. The data revealed that 17.8% of students’ relatives with COVID-19 presented no stress. These findings agree with the studies of Planchuelo et al., [56], who indicated that people who knew someone who had died of COVID-19 obtained lower scores for stress and anxiety. The students quarantined for COVID-19 proportionally had less stress than those who did not have to be isolated at home.

We must bear in mind that, although university students are not particularly concerned about their own health risks during the pandemic, they tend to worry about their family’s health, which could be high in Hispanic culture in which both strong family and interdependent relationships prevail [57].

The herein employed scales conferred reliability to this study because Cronbach’s alpha obtained a score over 0.7. In this study, PSS-10-C was 0.789, which is similar to other studies conducted in the general population with 0.86, and also with 0.82 [28,29,30], where 52% of them were university students [31]. Cronbach’s alpha for GADS anxiety and depression was 0.794 vs. 0.752, which are similar results to those reported in different studies performed with students [58,59].

## 5. Conclusions

Our findings suggest that stress from the COVID-19 pandemic could affect females and last-year students, those who do not work and those who had come into less contact with COVID-19. The students who perceived more stress were more likely to suffer anxiety and depression.

### Implications for Research and Interventions

The present study was conducted when most universities had resumed face-to-face classes, especially studies related to Health Sciences with plenty of teaching placements. This teaching was performed with certain precautions to avoid another outbreak; e.g., forming small groups in class, social distancing, ventilation, cleaning, using masks, etc. Some students might perceive this step as getting back to normal. Having acquired more information about the pandemic, students may feel less uncertainty and voice fewer concerns. Others, however, as our results reflect, may present symptoms of stress, anxiety and depression that persist. The incorporation of a lean methodology may lead to the identification and elimination of waste in teaching-learning processes [60]. Therefore, psychological support measures for students in these groups should be prioritized.

Our study is not without its limitations, which must be considered. First, it is an observational study and all the subjects voluntarily offered to participate. This could represent a selection bias. When our self-administered questionnaire was sent, students had to complete many academic surveys to evaluate the teaching and teachers of the first semester. Therefore, it is feasible to believe that many more students would have completed our questionnaire if they had received it at another time. Another limitation was that this study was conducted with a university population related to health and attending to patients, which means that the extrapolation of these results cannot be directly applied to all university students or to the general population.

## Figures and Tables

**Table 1:** Study sample’s socio-demographic and well-being/health characteristics.

Variables.	Total (*n* = 252)
	Mean (SD)
Age	21.02 (5.20)
	Frequencies (%)
Gender Male Female	46 (18.3%) 206 (81.7%)
Country Spain Elsewhere	232 (92.1%) 20 (7.9%)
Place of Residence Rural Urban	64 (25.4%) 188 (74.6%)
Economic situation Grant-holder Depend on family income Independent Others	34 (13.5%) 197 (78.2%) 17 (6.7%) 4 (1.6%)
Smoking Ex-smoker Occasional smoker No Yes	15 (6%) 25 (9.9%) 183 (72.6%) 29 (11.5%)
Chronic disease No Yes	213 (84.5%) 39 (15.5%)
First year Nursing Physiotherapy Occupational therapy	22 (8.7%) 22 (8.7%) 51 (20.2%)
Second year Nursing Physiotherapy Occupational therapy	11 (4.4%) 31 (12.3%) 12 (4.8%)
Third year Nursing Physiotherapy Occupational therapy	23 (9.1%) 13 (5.2%) 8 (3.2%)
Fourth year Nursing Physiotherapy Occupational therapy Missing	19 (7.5%) 15 (6%) 23 (9.1%) 2 (0.8%)
Occupational status Do not work Others Working fulltime Working part-time Unpaid work	171 (67.9%) 12 (4.8%) 17 (6.7%) 30 (11.9%) 22 (8.8%)
Stress (PSS-10-C) No Yes	219 (86.9%) 33 (13.1%)
Anxiety (GADS) No Yes Missing	68 (27%) 180 (71.4%) 4 (1.6%)
Depression (GADS) No Yes Missing	47 (18.7%) 204 (81%) 1 (0.4)
Confined by COVID No Yes	140 (55.6%) 112 (44.4%)
Had COVID No Yes	226 (89.7%) 26 (10.3%)
Relative had COVID No Yes	202 (80.2%) 50 (19.8%)

**Table 2:** Characteristics of the study population’s stress and anxiety/depression.

	Stress (PSS-10-C)	Anxiety (GADS)	Depression (GADS)
Minimum Maximum	1 37	0.00 9	0.00 9
Mean (SD); 95% CI	17.91 (6.27)	5.14 (2.64)	3.66 (2.39)
Cronbach’s alpha	0.789	0.794	0.752
Stress (PSS-10-C)	1000	0.443 *	0.437 *
Anxiety (GADS)	0.443 *	1000	0.593 *
Depression (GADS)	0.437 *	0.593 *	1.000

* Correlation is significant at the 0.01 level (bilateral).

**Table 3:** Association between stress and socio-demographic variables.

	No Stress	Stress	Total (*n* = 252)	*p* Value (X^2^ Test)
Gender Male Female	43 (19.6%) 176 (80.4%)	3 (9.1%) 30 (90.9%)	46 (18.3%) 206 (81.7%)	0.144
First year Nursing Physiotherapy Occupational therapy	19 (22.1%) 18 (20.9%) 49 (57%)	3 (33.3%) 4 (44.4%) 2 (22.2%)	22 (23.2%) 22 (23.2%) 51 (53.7%)	0.121
Second year Nursing Physiotherapy Occupational therapy	10 (21.3%) 29 (61.7%) 8 (17%)	1 (14.3%) 2 (28.6%) 4 (57.1%)	11 (20.4%) 31 (57.4%) 12 (22.2%)	0.057
Third year Nursing Physiotherapy Occupational therapy	19 (55.9%) 12 (35.3%) 3 (8.8%)	4 (40%) 1 (10%) 5 (50%)	23 (52.3%) 13 (29.5%) 8 (18.2%)	0.010
Fourth year Nursing Physiotherapy Occupational therapy Missing	15 (26.3%) 14 (28%) 21 (42%)	4 (57.1%) 1 (14.3%) 2 (28.6%)	19 (33.3%) 15 (26.3%) 23 (40.4%) 2 (0.8%)	0.355
Occupational status Do not work Working fulltime Working part-time Doing unpaid work Others	144 (65.8%) 16 (7.3%) 29 (13.2%) 19 (8.7%) 11 (5%)	27 (81.8%) 1 (3%) 1 (3%) 3 (9.1%) 1 (3%)	171 (67.9%) 17 (6.7%) 30 (11.9%) 22 (8.8%) 12 (4.8%)	0.038
Anxiety (GADS) No Yes Missing	66 (30.1%) 150 (68.5%)	2 (6.1%) 30 (90.9%)	68 (27%) 180 (71.4%) 4 (1.6%)	0.013
Depression (GADS) No Yes Missing	44 (20.1%) 174 (79.5%)	3 (9.1%) 30 (90.9%)	47 (18.7%) 204 (81%) 1 (0.3%)	0.290
Relatives with COVID-19 No Yes	180 (82.2%) 39 (17.8%)	22 (66.7%) 11 (33.3%)	202 (80.2%) 50 (19.8%)	0.037

**Table 4:** Binary logistic regression.

Variables			95% CI for EXP (B)	
	Sig	OR (B)	Lower	Higher
Third year of Nursing	0.031	0.148	0.026	0.842
Third year of Physiotherapy	0.303	0.334	0.041	2.693
Third year of Occupational Therapy	0.271	0.200	0.011	3.527
Occupational status (do not work)	0.077	2.641	0.901	7.743
Anxiety (GADS)	0.000	1.504	1.228	1.842
Family relative with COVID-19	0.042	0.375	0.146	0.967

## Data Availability

The data presented in this study are available on request from the corresponding author. The data are not publicly available due to specific requirements from the clinical research ethics committee that reviewed and approved this investigation.

## References

[1] Wang C., Horby P.W., Hayden F.G., Gao G.F. (2020). A novel coronavirus outbreak of global health concern. Lancet.

[2] Adhikari S.P., Meng S., Wu Y.-J., Mao Y.-P., Ye R.-X., Wang Q.-Z., Sun C., Sylvia S., Rozelle S., Raat H. (2020). Epidemiology, causes, clinical manifestation and diagnosis, prevention and control of coronavirus disease (COVID-19) during the early outbreak period: A scoping review. Infect. Dis. Poverty.

[3] Vizcaya-Moreno M.F., Pérez-Cañaveras R.M. (2020). Social media used and teaching methods preferred by generation z students in the nursing clinical learning environment: A cross-sectional research study. Int. J. Environ. Res. Public Health.

[4] Ramos-Morcillo A.J., Leal-Costa C., Moral-García J.E., Ruzafa-Martínez M. (2020). Experiences of nursing students during the abrupt change from face-to-face to e-learning education during the first month of confinement due to COVID-19 in Spain. Int. J. Environ. Res. Public Health.

[5] Romero-Blanco C., Rodríguez-Almagro J., Onieva-Zafra M.D., Parra-Fernández M.L., Prado-Laguna M.D.C., Hernández-Martínez A. (2020). Physical activity and sedentary lifestyle in university students: Changes during confinement due to the covid-19 pandemic. Int. J. Environ. Res. Public Health.

[6] Ahmed N.J., Alrawili A.S., Alkhawaja F.Z. (2020). The Anxiety and Stress of the Public during the Spread of Novel Coronavirus (COVID-19). J. Pharm. Res. Int..

[7] Wang C., Pan R., Wan X., Tan Y., Xu L., Ho C.S., Ho R.C. (2020). Immediate psychological responses and associated factors during the initial stage of the 2019 coronavirus disease (COVID-19) epidemic among the general population in China. Int. J. Environ. Res. Public Health.

[8] González-Sanguino C., Ausín B., Castellanos M.A., Saiz J., Muñoz M. (2021). Mental health consequences of the Covid-19 outbreak in Spain. A longitudinal study of the alarm situation and return to the new normality. Prog. Neuro-Psychopharmacol. Biol. Psychiatry.

[9] Ozamiz-Etxebarria N., Dosil-Santamaria M., Picaza-Gorrochategui M., Idoiaga-Mondragon N. (2020). Niveles de estrés, ansiedad y depresión en la primera fase del brote del COVID-19 en una muestra recogida en el norte de España. Cad. Saude Publica.

[10] Baenas I., Caravaca-Sanz E., Granero R., Sánchez I., Riesco N., Testa G., Vintró-Alcaraz C., Treasure J., Jiménez-Murcia S., Fernández-Aranda F. (2020). COVID-19 and eating disorders during confinement: Analysis of factors associated with resilience and aggravation of symptoms. Eur. Eat. Disord. Rev..

[11] Odriozola-González P., Planchuelo-Gómez Á., Irurtia M.J., de Luis-García R. (2020). Psychological effects of the COVID-19 outbreak and lockdown among students and workers of a Spanish university. Psychiatry Res..

[12] Rodríguez-Rey R., Garrido-Hernansaiz H., Collado S. (2020). Psychological Impact and Associated Factors During the Initial Stage of the Coronavirus (COVID-19) Pandemic Among the General Population in Spain. Front. Psychol..

[13] Gómez-Salgado J., Andrés-Villas M., Domínguez-Salas S., Díaz-Milanés D., Ruiz-Frutos C. (2020). Related Health Factors of Psychological Distress During the COVID-19 Pandemic in Spain. Int. J. Environ. Res. Public Health.

[14] Mazza C., Ricci E., Biondi S., Colasanti M., Ferracuti S., Napoli C., Roma P. (2020). A Nationwide Survey of Psychological Distress among Italian People during the COVID-19 Pandemic: Immediate Psychological Responses and Associated Factors. Int. J. Environ. Res. Public Health.

[15] Brooks S.K., Webster R.K., Smith L.E., Woodland L., Wessely S., Greenberg N., Rubin G.J. (2020). The psychological impact of quarantine and how to reduce it: Rapid review of the evidence. Lancet.

[16] Krishnamoorthy Y., Nagarajan R., Saya G.K., Menon V. (2020). Prevalence of psychological morbidities among general population, healthcare workers and COVID-19 patients amidst the COVID-19 pandemic: A systematic review and meta-analysis. Psychiatry Res..

[17] Rajkumar R.P. (2020). COVID-19 and mental health: A review of the existing literature. Asian J. Psychiatr..

[18] Debowska A., Horeczy B., Boduszek D., Dolinski D. (2020). A repeated cross-sectional survey assessing university students’ stress, depression, anxiety, and suicidality in the early stages of the COVID-19 pandemic in Poland. Psychol. Med..

[19] Rotenstein L.S., Ramos M.A., Torre M., Bradley Segal J., Peluso M.J., Guille C., Sen S., Mata D.A. (2016). Prevalence of depression, depressive symptoms, and suicidal ideation among medical students a systematic review and meta-analysis. Jama.

[20] Eisenberg D., Hunt J., Speer N. (2013). Mental Health in American Colleges and Universities. J. Nerv. Ment. Dis..

[21] Al-Rabiaah A., Temsah M.-H., Al-Eyadhy A.A., Hasan G.M., Al-Zamil F., Al-Subaie S., Alsohime F., Jamal A., Alhaboob A., Al-Saadi B. (2020). Middle East Respiratory Syndrome-Corona Virus (MERS-CoV) associated stress among medical students at a university teaching hospital in Saudi Arabia. J. Infect. Public Health.

[22] Cerqueira J.J., Mailliet F., Almeida O.F.X., Jay T.M., Sousa N. (2007). The Prefrontal Cortex as a Key Target of the Maladaptive Response to Stress. J. Neurosci..

[23] Spector C., JFigueira J., Miramontes C., Canova-Barrios C. (2020). Enseñanza y evaluación a distancia en época de pandemia: Experiencia inicial de las Carreras de Salud de UCES. Rev. Argent. Educ. Médica.

[24] Luján Piedrahíta M. (2020). Virtualidad en el curso teórico de Medicina Interna en estudiantes de V, VI y VII semestre a propósito de la pandemia COVID-19 durante el primer semestre del 2020, Facultad de Medicina, Escuela de Ciencias de la Salud, Universidad Pontificia Bolivariana. Med. UPB.

[25] Chen J.C. (2014). Teaching nontraditional adult students: Adult learning theories in practice. Teach. High. Educ..

[26] Singh J., Matthees B., Odetunde A. (2021). Leaning online education during COVID-19 pandemic—Attitudes and perceptions of non-traditional adult learners. Qual. Assur. Educ..

[27] Melcher J., Hays R., Torous J. (2020). Digital phenotyping for mental health of college students: A clinical review. Evid. Based Ment. Health.

[28] Campo-Arias A., Pedrozo-Cortés M.J., Pedrozo-Pupo J.C. (2020). Pandemic-Related Perceived Stress Scale of COVID-19: An exploration of online psychometric performance. Rev. Colomb. Psiquiatr..

[29] Campo-Arias A., Bustos-Leiton G., Romero A. (2009). Consistencia interna y dimensionalidad de la Escala de Estrés Percibido (EEP-10 Y EEP-14) en una muestra de estudiantes universitarias de Bogotá. Colomb. Aquichan..

[30] Pedrozo-Pupo J.C., Pedrozo-Cortés M.J., Campo-Arias A. (2020). Perceived stress associated with COVID-19 epidemic in Colombia: An online survey. Cad. Saude. Publica.

[31] Remor E. (2006). Psychometric Properties of a European Spanish Version of the Perceived Stress Scale (PSS). J. Psychol..

[32] Aslan H., Pekince H. (2021). Nursing students’ views on the COVID-19 pandemic and their percieved stress levels. Perspect. Psychiatr. Care.

[33] Torun F., Torun S.D. (2020). The psychological impact of the COVID-19 pandemic on medical students in Turkey. Pak. J. Med. Sci..

[34] Rogowska A.M., Kuśnierz C., Bokszczanin A. (2020). Examining Anxiety, Life Satisfaction, General Health, Stress and Coping Styles During COVID-19 Pandemic in Polish Sample of University Students. Psychol. Res. Behav. Manag..

[35] Gavurova B., Ivankova V., Rigelsky M. (2020). Relationships between Perceived Stress, Depression and Alcohol Use Disorders in University Students during the COVID-19 Pandemic: A Socio-Economic Dimension. Int. J. Environ. Res. Public Health.

[36] De Araújo F.J., de Lima L.S.A., Cidade P.I.M., Nobre C.B., Neto M.L.R. (2020). Impact Of Sars-Cov-2 And Its Reverberation In Global Higher Education And Mental Health. Psychiatry Res..

[37] Sahu P. (2020). Closure of Universities Due to Coronavirus Disease 2019 (COVID-19): Impact on Education and Mental Health of Students and Academic Staff. Cureus.

[38] Molina J.D., Andrade-Rosa C., González-Parra S., Blasco-Fontecilla H., Real M.A., Pintor C. (2006). The factor structure of the General Health Questionnaire (GHQ): A scaled version for general practice in Spain. Eur. Psychiatry.

[39] Goldberg D., Bridges K., Duncan-Jones P., Grayson D. (1988). Detecting anxiety and depression in general medical settings. Br. Med. J..

[40] Cohen S., Kamarck T., Mermelstein R. (1983). A Global Measure of Perceived Stress. J. Health Soc. Behav..

[41] Ioannidis J.P.A., Axfors C., Contopoulos-Ioannidis D.G. (2021). Second versus first wave of COVID-19 deaths: Shifts in age distribution and in nursing home fatalities. Environ. Res..

[42] Bashir T.F., Hassan S., Maqsood A., Khan Z.A., Issrani R., Ahmed N., Bashir E.F. (2020). The Psychological Impact Analysis of Novel COVID-19 Pandemic in Health Sciences Students: A Global Survey. Eur. J. Dent..

[43] Pellegrino R., Viegi G., Brusasco V., Crapo R.O., Burgos F., Casaburi R.E.A., Coates A., Van Der Grinten C.P.M., Gustafsson P., Hankinson J. (2005). Interpretative strategies for lung function tests. Eur Respir. J..

[44] Huang Y., Zhao N. (2020). Generalized anxiety disorder, depressive symptoms and sleep quality during COVID-19 outbreak in China: A web-based cross-sectional survey. Psychiatry Res..

[45] Sánchez M.J.B., Islas C.L.R., Escobar I.V., Rico L.E.S. (2008). Symptoms of anxiety and depression in resident physicians at high risk of stress. Psiquiatr. Biol..

[46] Cao W., Fang Z., Hou G., Han M., Xu X., Dong J., Zheng J. (2020). The psychological impact of the COVID-19 epidemic on college students in China. Psychiatry Res..

[47] Tang W., Hu T., Hu B., Jin C., Wang G., Xie C., Chen S., Xu J. (2020). Prevalence and correlates of PTSD and depressive symptoms one month after the outbreak of the COVID-19 epidemic in a sample of home-quarantined Chinese university students. J. Affect Disord..

[48] Salman M., Asif N., Mustafa Z.U., Khan T.M., Shehzadi N., Tahir H., Salman M., Asif N., Mustafa Z., Khan T.M. (2020). Psychological Impairment and Coping Strategies During the COVID-19 Pandemic Among Students in Pakistan: A Cross-Sectional Analysis. Disaster Med. Public Health Prep..

[49] Özdin S., Bayrak Özdin Ş. (2020). Levels and predictors of anxiety, depression and health anxiety during COVID-19 pandemic in Turkish society: The importance of gender. Int. J. Soc. Psychiatry.

[50] Solomou I., Constantinidou F. (2020). Prevalence and predictors of anxiety and depression symptoms during the COVID-19 pandemic and compliance with precautionary measures: Age and sex matter. Int. J. Environ. Res. Public Health.

[51] Rodríguez-Rey R., Garrido-Hernansaiz H., Collado S. (2020). Psychological impact of COVID-19 in Spain: Early data report. Psychol. Trauma Theory Res. Pract. Policy.

[52] Lima-Serrano M., Martínez-Montilla J.M., Guerra-Martín M.D., Vargas-Martínez A.M., Lima-Rodríguez J.S. (2018). Factores relacionados con la calidad de vida en la adolescencia. Gac. Sanit..

[53] McLean C.P., Anderson E.R. (2009). Brave men and timid women? A review of the gender differences in fear and anxiety. Clin. Psychol. Rev..

[54] Ministerio de Salud (2017). Encuesta Nacional de Salud. Salud Mental. https://www.mscbs.gob.es/estadEstudios/estadisticas/encuestaNacional/encuestaNac2017/SALUD_MENTAL.pdf.

[55] Achdut N., Refaeli T. (2020). Unemployment and Psychological Distress among Young People during the COVID-19 Pandemic: Psychological Resources and Risk Factors. Int. J. Environ. Res. Public Health.

[56] Planchuelo-Gómez Á., Odriozola-González P., Irurtia M.J., de Luis-García R. (2020). Longitudinal evaluation of the psychological impact of the COVID-19 crisis in Spain. J. Affect Disord..

[57] Campos B., Kim H.S. (2017). Incorporating the cultural diversity of family and close relationships into the study of health. Am. Psychol..

[58] Luo Y., Meng R., Li J., Liu B., Cao X., Ge W. (2019). Self-compassion may reduce anxiety and depression in nursing students: A pathway through perceived stress. Public Health.

[59] Baran S., Teul-Swiniarska I., Dzieciolowska-Baran E., Lorkowski J., Gawlikowska-Sroka A. (2013). Mental Health of Polish Students and the Occurrence of Respiratory Tract Infections. Adv. Exp. Med. Biol..

[60] Singh J. (2019). The lean prescription for non-traditional adult learners. Qual. Assur. Educ..

